# Evolution and Identification of the WRKY Gene Family in Quinoa (*Chenopodium quinoa*)

**DOI:** 10.3390/genes10020131

**Published:** 2019-02-11

**Authors:** Hong Yue, Xi Chang, Yongqiang Zhi, Lan Wang, Guangwei Xing, Weining Song, Xiaojun Nie

**Affiliations:** 1 State Key Laboratory of Crop Stress Biology in Arid Areas and College of Agronomy, Northwest A&F University, Yangling 712100, China; yuehong@nwsuaf.edu.cn (H.Y.); zhiyqshaanxi@nwsuaf.edu.cn (Y.Z.); wanglan_@nwsuaf.edu.cn (L.W.); xinggw@nwsuaf.edu.cn (G.X.); wnsong@nwsuaf.edu.cn (W.S.); 2 College of Life Sciences, Northwest A&F University, Yangling 712100, China; 3 Xizang Agriculture and Animal Husbandry College, Linzhi 860000, China; changxi2007@yahoo.com

**Keywords:** WRKY, evolution, quinoa, abiotic stress, expression profiles

## Abstract

The WRKY gene family plays a unique role in plant stress tolerance. Quinoa is a cultivated crop worldwide that is known for its high stress tolerance. The WRKY gene family in quinoa has not yet been studied. Using a genome-wide search method, we identified 1226 WRKY genes in 15 plant species, seven animal species, and seven fungi species. WRKY proteins were not found in animal species and five fungi species, but were, however, widespread in land plants. A total of 92 CqWRKY genes were identified in quinoa. Based on the phylogenetic analysis, these CqWRKY genes were classified into three groups. The CqWRKY proteins have a highly conserved heptapeptide WRKYGQK with 15 conserved elements. Furthermore, a total of 25 CqWRKY genes were involved in the co-expression pathway of organ development and osmotic stress. The expression level of more than half of these CqWRKY genes showed significant variation under salt or drought stress. This study reports, for the first time, the findings of the CqWRKY gene family in quinoa at the genome-wide level. This information will be beneficial for our understanding of the molecular mechanisms of stress tolerance in crops, such as quinoa.

## 1. Introduction

The WRKY gene family is an important transcription factor, playing a unique regulatory role in plants’ defense responses to abiotic and biotic stresses. They can positively or negatively regulate the expression of other genes to increase the stress tolerance of plants [1]. In wild diploid woodland strawberry, the expression of *FvWRKY42* was induced and interacted with different stress-response proteins under powdery mildew, salt, drought, salicylic acid (SA), methyl jasmonate, abscisic acid (ABA) and ethylene (ET) treatment [2]. The overexpression of *FvWRKY42* in *Arabidopsis* resulted in the enhanced powdery mildew resistance, salt and drought stress tolerance [2]. Previous study also found that *VvWRKY30* in grape had a positive effect on salt stress and transgenic *Arabidopsis* had super salt stress resistance by regulating a series of glycol-metabolism dependent genes [3]. *ZmWRKY40* and *ZmWRKY106* were induced in maize by salt, drought, ABA and high temperatures. Overexpression of *ZmWRKY40* regulates the diverse stress-related genes, including *STZ*, *DREB2B* and *RD29A* in *Arabidopsis* and rice, to improve drought stress tolerance, and overexpression of *ZmWRKY106* regulates ABA-signaling pathway related genes to improve the drought and heat tolerance compared to wild type, respectively [4,5]. What is more, GmWRKY27 can interact with GmMYB174 and also suppresses *GmNAC29* expression. Overexpression and knockout of *GmWRKY27* demonstrated that it played crucial roles in response to salt and drought stress in soybean [6]. The WRKY transcription factor also plays vital roles in regulating grain yield in transgenic crops. For example, Gao et al., reported that overexpression of *TaWRKY2* could significantly enhanced grain yield in transgenic wheat, which had longer panicle length and more kernels per spike contrast to wild type [7]. In rice, knockout of *OsWRKY47* displayed lower drought tolerance and reduced yield, while overexpressing of *OsWRKY47* increased the drought tolerance [8]. The tiller number, grain weight and P concentration were also increased in *OsWRKY74* overexpressing plants in contrast to WT under P-deficient stress [9].

Generally, the WRKY proteins possess 60 amino acid domains known as WRKY domains. These domains, together with the zinc-finger motif (Cys2-His2) at the C-terminal, act as a specific DNA-binding peptide sequence WRKYGQK at the N-terminal. The WRKY domains interact specifically with a DNA motif termed the W-box (TGACC(A/T)) or SURE (cis-responsive element) in the target gene promoter, then regulates their biotic and abiotic stress responses [10,11]. Based on the sequence features of the zinc-finger motif and number of WRKY domains, WRKY genes were further classed into three groups: WRKY I, II, and III. Members of group I have the C2H2 (C–X4–C–X22–23–HXH) zinc finger motif and two WRKY domains. Members of group II have a similar zinc finger motif to group I, but only have one WRKY domain. All members of group III contain the C2CH (C-X7-C-X23-HXC) motif and one WRKY domain [12,13]. Group II can be further divided into five subgroups: II-a, II-b, II-c, II-d, and II-e. The WRKY gene families play a unique role in regulating developmental and multiple physiological processes. For instance, in *Arabidopsis*, the suppression of *WRKY75* expression, and lateral root number and root length were increased significantly when under abiotic stresses [14]. Similarly, the group I WRKY gene, *GhWRKY25* was cloned from cotton, and the expression level of *GhWRKY25* was reduced under abiotic stresses. Overexpression of *GhWRKY25* not only enhanced sensitivity to fungal pathogen B. cinerea in *Nicotiana benthamiana* by reducing the expression of SA or ET signaling related genes, but also reduced tolerance to stress caused by drought [15]. The expression level of *VfWRKY1* and *VfWRKY2* was significantly increased in various plant organs of faba bean when under drought or salt stress [16]. Furthermore, *CsWRKY2* in tea (*Camellia sinensis* L.) was up-regulated by cold or drought stress, which suggested that CsWRKY2 plays an important role in the response to cold or drought stress by participating in the ABA signaling pathway [17].

WRKY II and III proteins play important roles in leaf senescence and plant stress response. Two WRKY transcription factors, WRKY6 and WRKY11, have been shown to play a role in leaf senescence of *Arabidopsis* [14]. In comparison, WRKY III members, WRKY54 and WRKY70, act as negative regulators of leaf senescence [18]. Overexpression of *MtWRKY76* in cotton in rice have also been reported to enhance their salt and drought tolerance [19]. Furthermore, WRKY III proteins can bind to cis elements in the promoter regions of other genes to enhance their expression in response to pathogenic stresses [20].

Quinoa (*Chenopodium quinoa* Wild.) is one of the world’s oldest cultivated crops. It was first domesticated as the staple food in South America more than 7000 years ago [21,22]. The grains of quinoa are high in nutritional value for humans and animals. The protein, total fat and dietary fiber contents ranged from 9.1 g to 15.7 g, 4.0 g to 7.6 g, 8.8 g to 14.1 g per 100 g of fresh quinoa grain, respectively [23,24].

The sequence of the plant whole genome is useful to identify gene families, and WRKY proteins have been widely identified in diverse plant species [25,26]. There are 102 WRKY genes found in rice, as well as 32 in broomcorn millets and 77 peanut strains by using HMMER search program [1,6,27]. However, to date, the role/function of the WRKY gene family is poorly understood in quinoa. Here, we systematically characterized the WRKY gene family in quinoa by genome-wide search, and identifiedthe evolutionary relationship of CqWRKY with other plant WRKYs. The phylogeny evolutionary relationships, conserved motifs, subgroup classification, regulatory network, and the expression patterns of WRKY proteins were analyzed. These results will be beneficial for our understanding of the molecular mechanisms of stress tolerance in crops such as quinoa. These results will not only provide the potential candidate for further functional analysis, but also be beneficial for our understanding of the molecular mechanisms of stress tolerance in quinoa and beyond.

## 2. Materials and Methods 

### 2.1. Identification of WRKY Gene Family and Chromosomal Localizations

Animal and fungi protein sequences were downloaded from NCBI, plant protein sequences were downloaded from Ensembl plants (http://plants.ensembl.org/index.html), thequinoa genome and protein sequences were downloaded from the Phytozome database (http://www.phytozome.net/) and NCBI, and the WRKY protein sequences were screened to identify the WRKY genes [21,28]. These protein sequences were firstly used to create the local database. The database was then searched against known WRKY protein sequences collected from *Arabidopsis*. We used the local BLASTP program (https://blast.ncbi.nlm.nih.gov) with an E-value cut-off <1e –5 in our database search [29]. After manual curation, the hidden Markov model (HMM) profile of the conserved WRKY domain (PF03106) sequences were obtained from the PFAM 31.0 database(http://pfam.xfam.org/) and the HMM 3.2 software (http://hmmer.org/) was used to search all WRKY proteins from the 15-plant species, 7 fungi species, and 7 animal species [30]. Redundant sequences and incomplete residual sequences were removed from protein sequences containing complete WRKY domains by using DNAMAN 5.0 (LynnonBioSoft, Quebec, Canada). The molecular weight, amino acid length, and isoelectric point were computed for each WRKY in quinoa by using the ExPASy online database. Cello v2.5 software, pLoc-mPlant (http://www.jci-bioinfo.cn/pLoc-mPlant/) and PSORT (https://psort.hgc.jp/) were used to predict the subcellular localization of these CqWRKY proteins. All parameters were set as the default. The genome locations were mapped on scaffolds and chromosomes of the quinoa genome by using the location BLASTN + 2.8.1 program (NCBI, Bethesda, MD, USA) with the E-value <10−5 and the best hits were obtained [21].

### 2.2. Phylogenetic Analysis, Gene Structure, Protein Conserved Motifs Identification and Gene Duplication

The ClutsalW 1.83 program was used for multiple protein sequence alignments between the plants, fungi, and animals. MEGA6.0 (Phoenix, AZ, USA) was used to construct the phylogenetic tree with the neighbor-joining (NJ) method with 1000 bootstrap replications.

In quinoa, conserved motifs of CqWRKYs were investigated using MEME (Multiple EM for Motif Elicitation, http://meme-suite.org/tools/meme) with the following parameter setup: number of repeat elements was set to any, maximum motifs were set to 15, and the best motif widths were set to 6–200 residues [31]. The gene structure information including intron, exon, and genome location of the CqWRKY genes were obtained from the quinoa genome database and displayed in the GSDS (Gene Structure Display Server, http://gsds.cbi.pku.edu.cn/) [32]. Gene duplication events of *WRKY* genes in quinoa were investigated as described by Wang et al. By using three criteria: the alignment covered >80% of the longer gene, the aligned region had an identity >80% and the tightly linked genes was counted as one duplication event [33]. Then, the duplicated regions were visualized by the Circos tool (http://circos.ca/) in the quinoa genome.

### 2.3. Interaction Network of CqWRKY Genes

To examine the regulatory role of CqWRKY genes, interaction regulation networks of CqWRKYs with other quinoa genes wereconstructed based on the orthologous comparison between *Arabidopsis* and quinoa. The STRING v10.5 (http://string-db.org/) and AraNet V2 (https://www.inetbio.org/aranet/) tools were used to analyze the *Arabidopsis* orthologous gene [34]. Cytoscape v3.2.1 (San Diego, CA, USA) and BiNGO 3.0.3 (https://www.psb.ugent.be/cbd/papers/BiNGO/Home.html) were used to map the CqWRKY proteins and to construct biological interaction pathways of the specific gene sets [35].

### 2.4. Expression Profile Analysis in Various Tissues and under Different Abiotic Stresses of TaHDZ Genes

Publicly available quinoa RNA-Seq datasets were obtained from the SRA (Sequence Read Archive, https://www.ncbi.nlm.nih.gov/sra). These data were analyzed for the expression patterns of the identified CqWRKY genes. Materials from dry seed (SRS2464892), 1-week seedling (SRS2434487, SRS2464890), leaf (SRS4026093, SRS4026094, SRS4026095), stem (SRS2464891), and inflorescence (SRS2464889) were used to identify the tissue specific expression profiles. Moreover, salt (SRS2458686, SRS2458681, SRS2458685) and drought (SRS1204205) stress treatments were used to examine the stress response genes. The expression level was normalized and log10-transformed were used for producing the heat map using the heatmap Development Package in R software 3.5.1 (https://www.r-project.org/) [36]. 

## 3. Results

### 3.1. Global Identification and Evolution of WRKY Proteins from Eukaryotes to Plants

**Figure 1:**
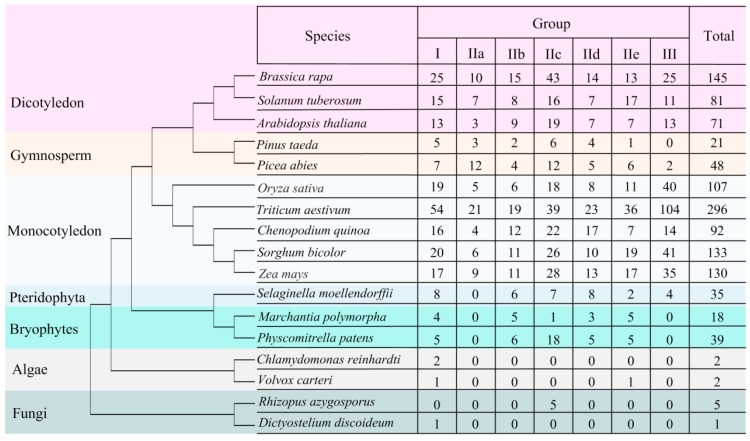
Species phylogeny and numbers of WRKY genes in each species.

To understand the evolutionary history and relationship of WRKY, we identified 1226 WRKY genes using profile HMM searches and BLASTP of 15 representative plant species, 7 animal species, and 7 fungi species (Table A1, Supplementary Material 1). The results showed that WRKY proteins were not identified in the 7 animal species including flatworms, molluscs, horsehair worm, fish, amphibians, and reptiles. Furthermore, no WRKY werefound from five of the fungi species. However, five and one WRKY proteins were identified in *Rhizopus azygosporus* and *Dictyostelium discoideum*, respectively. Five WRKY proteins from *Rhizopus azygosporus* had one WRKY domain, and one WRKY protein from *Dictyostelium discoideum* had two WRKY domains. This phenomenon was also found in Giardia lamblia [37]. A total of 15 plant species including three dicotyledon species, two gymnosperm species, five monocotyledon species, one pteridophyta species, two bryophytes species, and two algae, were used to investigate WRKY. The results showed that WRKY were widespread in land plants. Among them, angiosperm plants showed the most abundant WRKY genes, followed by gymnosperm species, pteridophyta species, and bryophytes species. In particular, a total of 296 WRKY genes were found in wheat. This difference between these species of mainly the WRKY gene family did expand with wheat polyploidization and genome evolution. The number of WRKY genes in algae was particularly low when compared to that of other plant species. Interestingly, the evolution of *Chlamydomonas reinhardtii* was before the divergence of land plants, so we found that only two WRKY proteins were identified and classed into group I. At the same time, two WRKY proteins were found in *Volvox carteri* (Figure 1). This phenomenon may have happened due to algae with only a single copy, so the WRKY gene duplication events occurred during the evolution from lower to higher plants [37].

**Figure A1:**
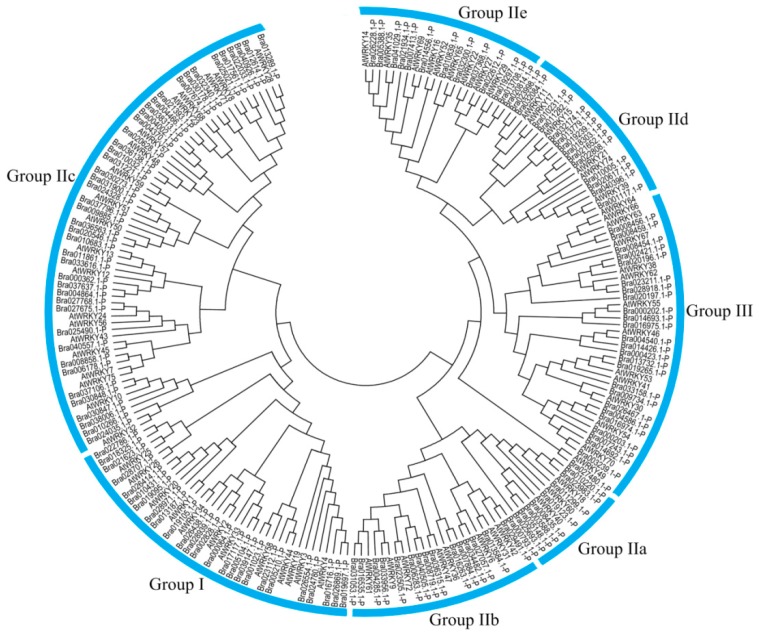
Subgroup classification of the *Brassica rapa* WRKYs.

**Figure A2:**
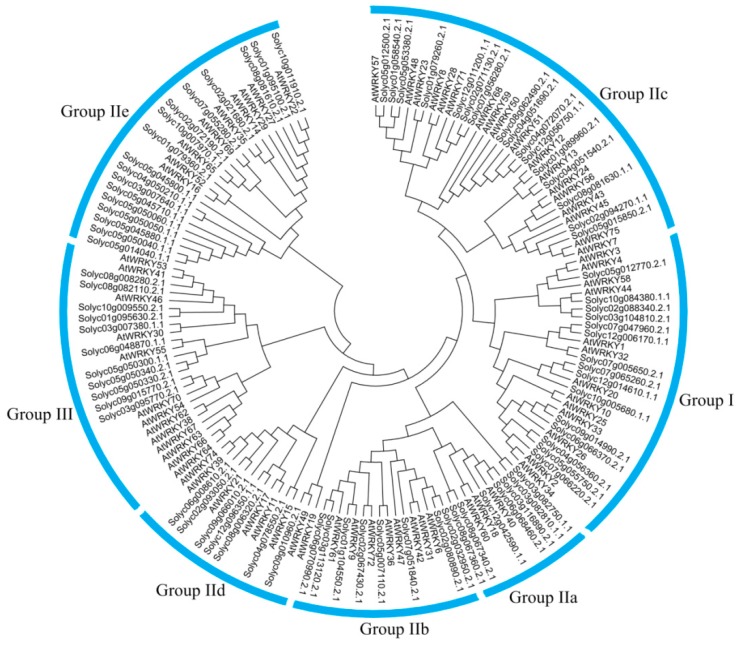
Subgroup classification of the *Solanum lycopersicum* WRKYs.

**Figure A3:**
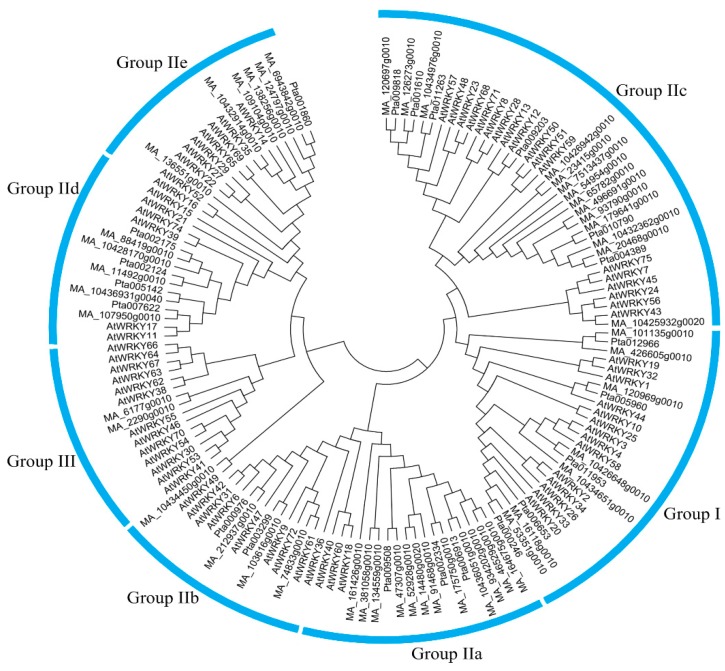
Classification of the gymnosperm WRKYs.

**Figure A4:**
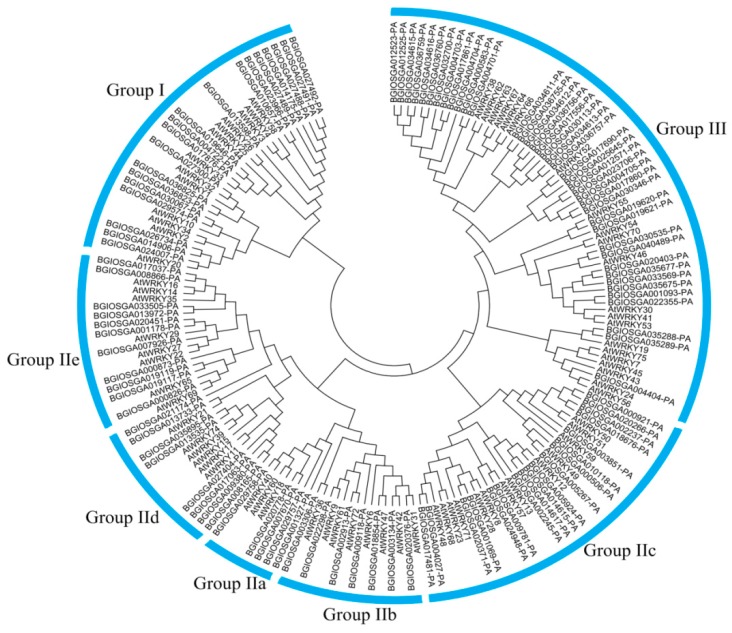
Classification of the *Oryzasativa* WRKYs.

**Figure A5:**
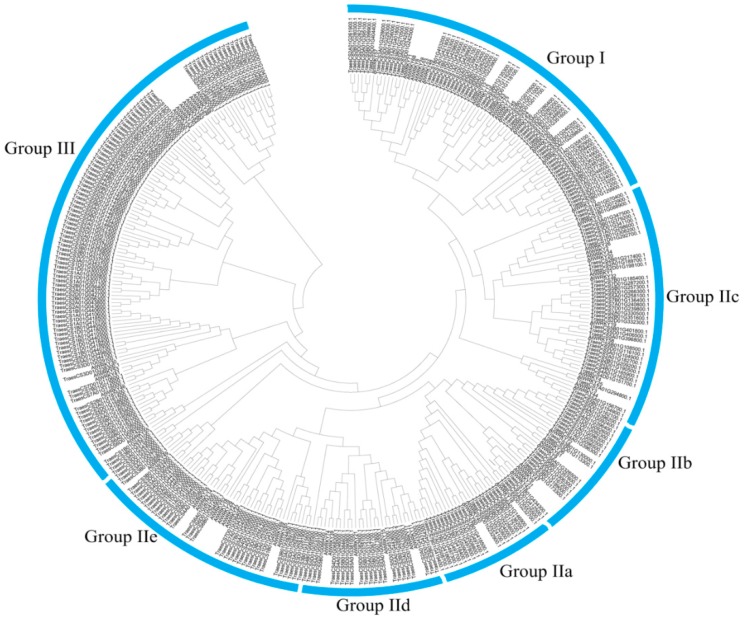
Classification of the *Triticum aestivum* WRKYs.

**Figure A6:**
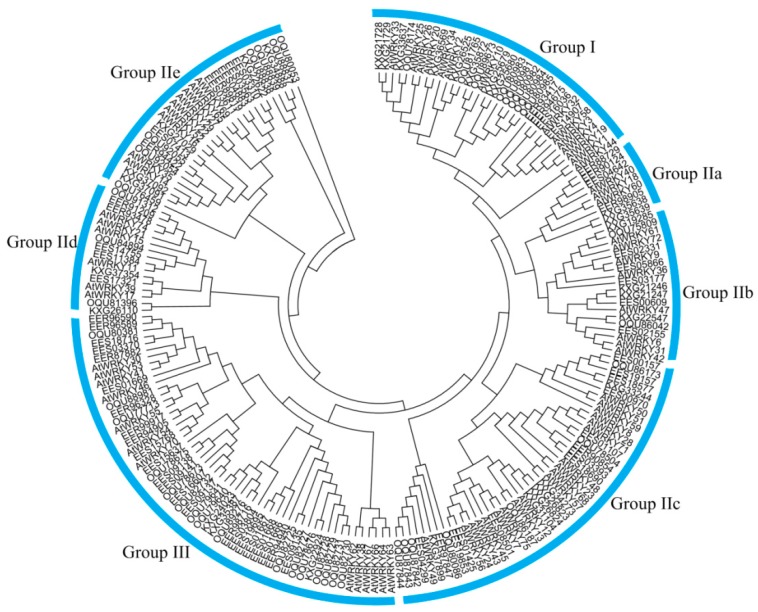
Classification of the *Sorghum bicolo* WRKYs.

**Figure A7:**
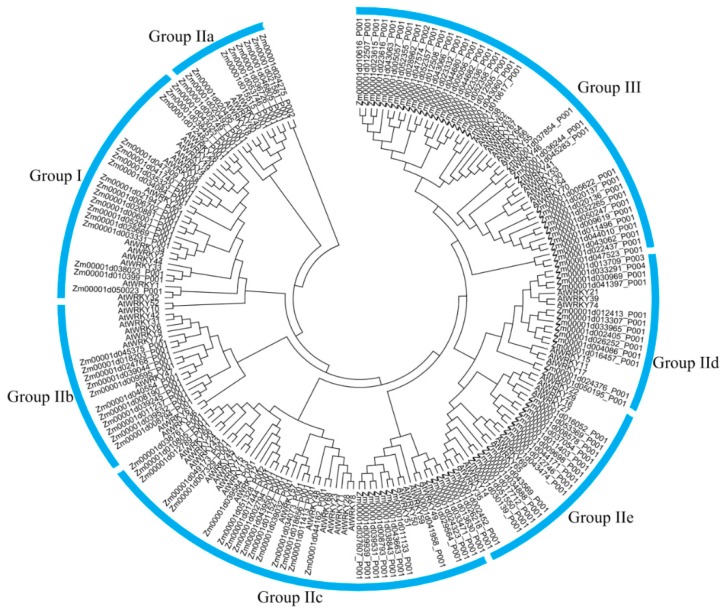
Classification of the *Zeamays* WRKYs.

**Figure A8:**
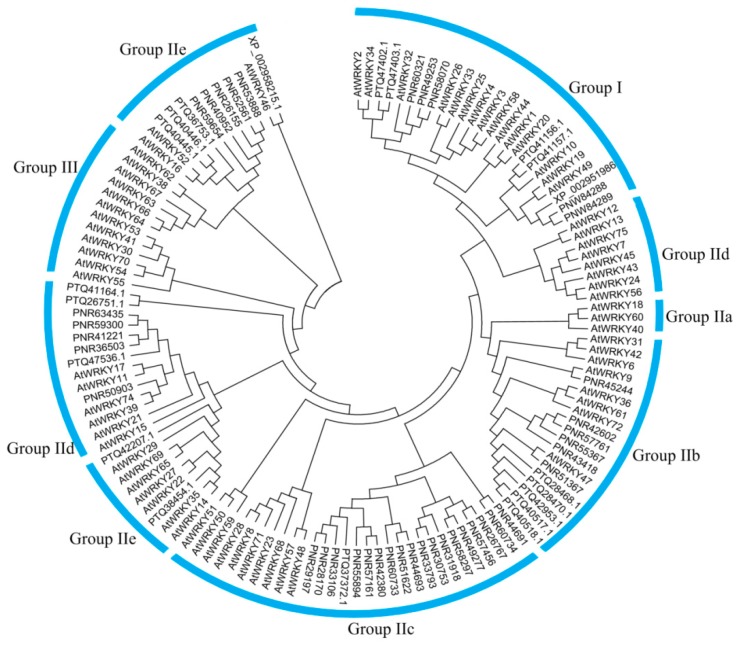
Classification of the bryophytes and algae WRKYs.

**Figure A9:**
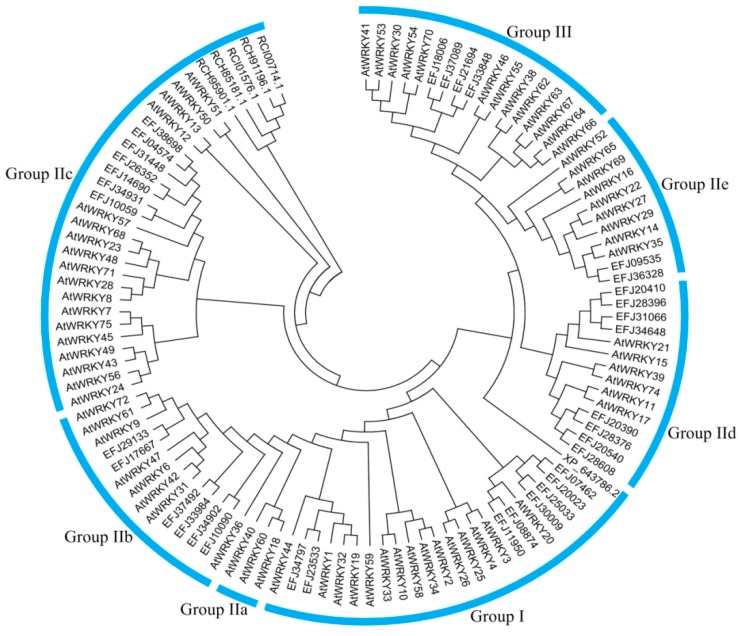
Classification of the fungi and pteridorphyta WRKYs.

Additionally, the number of WRKY in each subgroup of these species was investigated (Figure A1, Figure A2, Figure A3, Figure A4, Figure A5, Figure A6, Figure A7, Figure A8 and Figure A9). In monocotyledon species, group III was the larger subgroup except for quinoa while group II-c was the larger subgroup in dicotyledon and gymnosperm species except for *Solanum tuberosum*. Group II-a was present only in angiosperm and gymnosperm, but not from ancient plants, and group III arose early in the evolution of pteridophyta plants and was conserved across angiosperm, gymnosperm, and pteridophyta (Figure 1).

### 3.2. Identification of WRKY Genes in Quinoa

A total of 92 non-redundant genes containing the complete WRKY domains were identified. These protein sequences were named as CqWRKY1A to CqWRKY58A and regarded as the putative quinoa WRKY genes. These WRKY genes had large compositional differences. The molecular weights of these genes ranged from 21.8 kDa to 91.0 kDa, their length from 191 to 639 amino acids, and the isoelectric points were from 5.3 to 9.8. Among these proteins, 86 CqWRKY were located in the nuclear, only CqWRKY30A-2 and CqWRKY51A-1 in the extracellular, CqWRKY47B-1/2 in the peroxisome, CqWRKY54 in the chloroplast and CqWRKY58A in the cytoplasm, respectively. 92 CqWRKY genes were unevenly distributed on all the 18 quinoa chromosomes, of which chromosome 7A contained the most CqWRKY genes with the number of 18, followed by 1B with the number of 16, then 10B with the number of 11, while the chromosome 3B had not CqWRKY gene. In total, 38 and 40 CqWRKY were located on the A and B sub-genome, respectively (Table A2).

### 3.3. Multiple Sequence Alignment of CqWRKY

**Figure A10:**
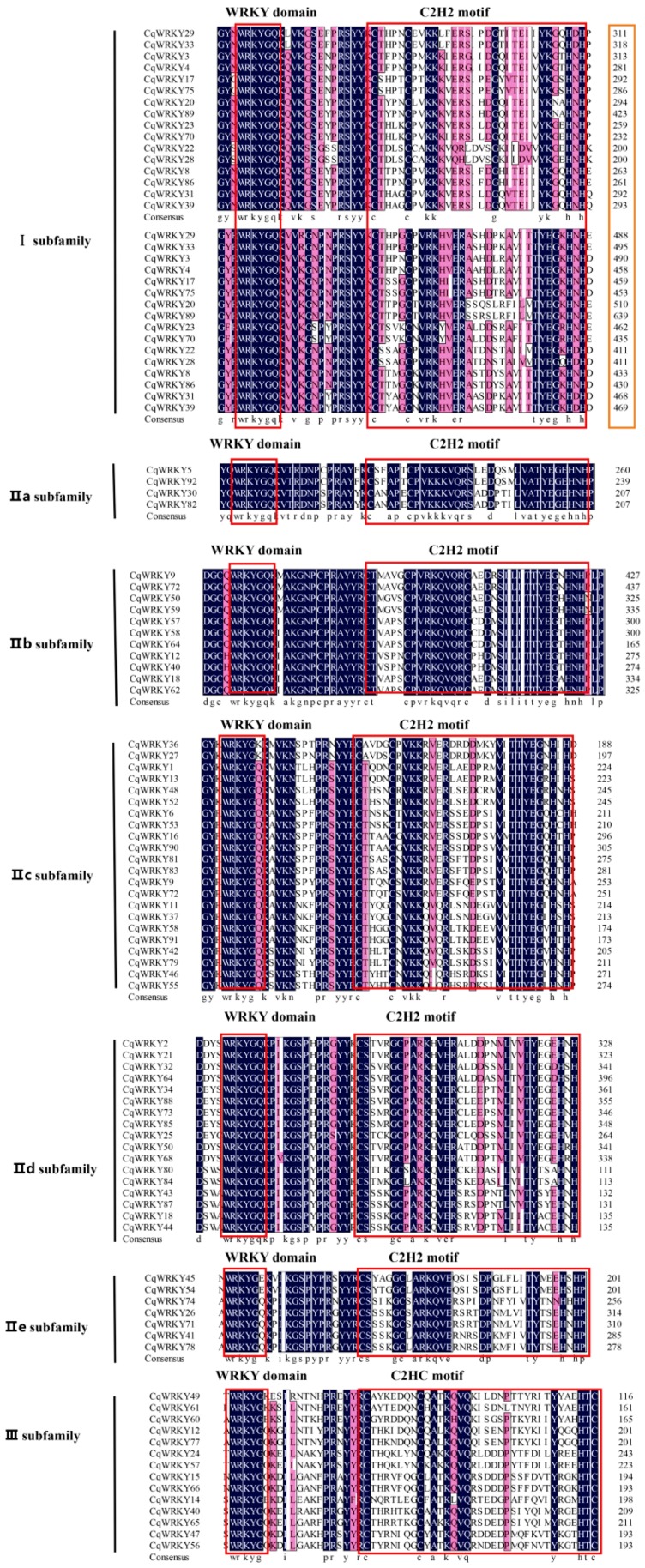
Alignment of the CqWRKY in quinoa. Conserved amino acids are shown by the blank background, WRKY domains and zinc-finger motifs are represented by the red box.

All CqWRKY proteins were classified into three groups. Group I was comprised of 16 CqWRKY proteins, each containing two WRKY domains and C2H2 zinc-finger motif. Group II was comprised of 62 proteins, each with one WRKY domain and a similar zinc-finger motif. Group III was comprised of 14 proteins, each containing only one WRKY domain and a C2CH zinc-binding motif. Seven CqWRKY proteins had a difference by a single amino acid in the WRKY domain. Four CqWRKY proteins including CqWRKY30A-1 and CqWRKY30A-2 of group II and CqWRKY51A-1 and CqWRKY51A-3 of group III contained the common variant sequence WRKYGKK. Three CqWRKY proteins, CqWRKY44A, CqWRKY45B, and CqWRKY51A-2, had the less common variant sequence WRKYGEK. The remaining 85 CqWRKY proteins had the highly conserved sequence WRKYGQK (Figure A10).

### 3.4. Phylogenetic Analysis of CqWRKY Genes

**Figure 2:**
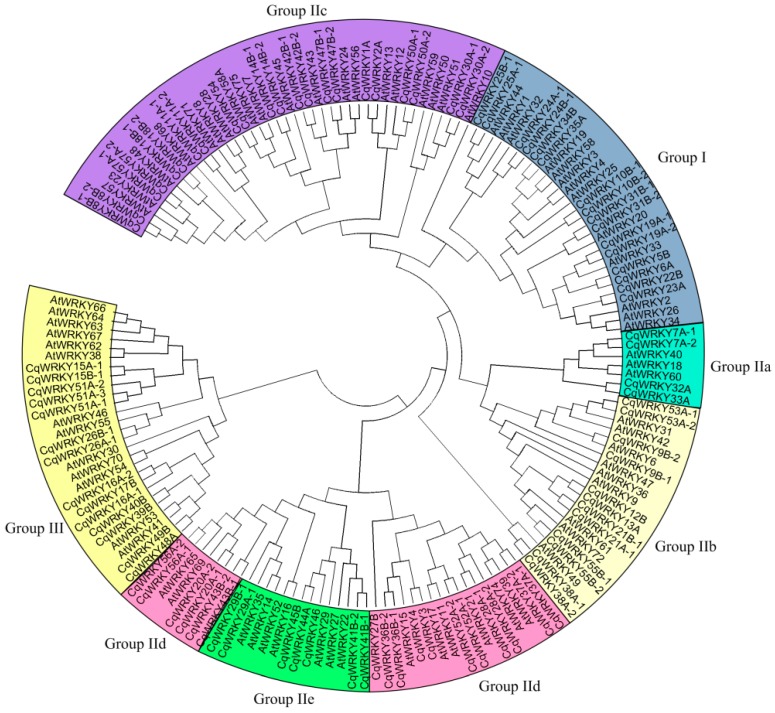
Phylogenetic analysis of WRKY proteins among quinoa and *Arabidopsis*.

A previous study reported that the WRKY transcription factor family had an early origin in eukaryotes, where this ancestral gene seems to have duplicated many times during the evolution of plants, resulting in a large gene family for WRKY proteins. However, the WRKY gene family was highly conserved during the evolution process in plants, regardless of being in monocots or eudicots, which could be classified into three groups according to the WRKY motif and zinc-finger sequence [37]. In order to explore the phylogenetic relationships of the WRKY gene family in quinoa, the 92 CqWRKY proteins of quinoa and 72 available from *Arabidopsis* were selected for phylogenetic analysis. Using the same classification criteria as in *Arabidopsis*, the WRKY proteins of quinoa were classified into three groups, I, II, and III, containing 16, 62, and 14 CqWRKY proteins, respectively. Group II was further classified into five sub-groups, from II a–e, containing 4, 12, 22, 17, and 7 CqWRKY proteins, respectively (Figure 2). Group II proteins were the most abundant type in quinoa, accounting for 67.4% of all CqWRKY proteins. It was similar to that of *Arabidopsis* with a percentage of 65.2%. This phenomenon was also found in peanut and sesame [6,38]. However, group III was the largest group of the WRKY gene family in broomcorn millet and wheat, which comprised about 50% and 41%, respectively [1,39]. Generally, group-III members accounted for 20% of the WRKY gene family in higher plants [40]. The WRKY genes from quinoa and *Arabidopsis* showed an interspersed distribution in groups I–III suggesting that the expansions of WRKY occurred before the divergence of the two species. Furthermore, group I members of the WRKY genes are the most ancient, with loss or gain of the N-terminal domain during the evolution process, and group II/III evolved late in land plants that contained only the C-terminal domain [41]. Furthermore, the number of CqWRKY proteins in group II-c was higher than the other subgroups. Group II-c was shown to be relatives of group I in quinoa, and this phenomenon was also found in wheat [42].

### 3.5. Conserved Motifs and Protein Structure Analysis

**Figure 3:**
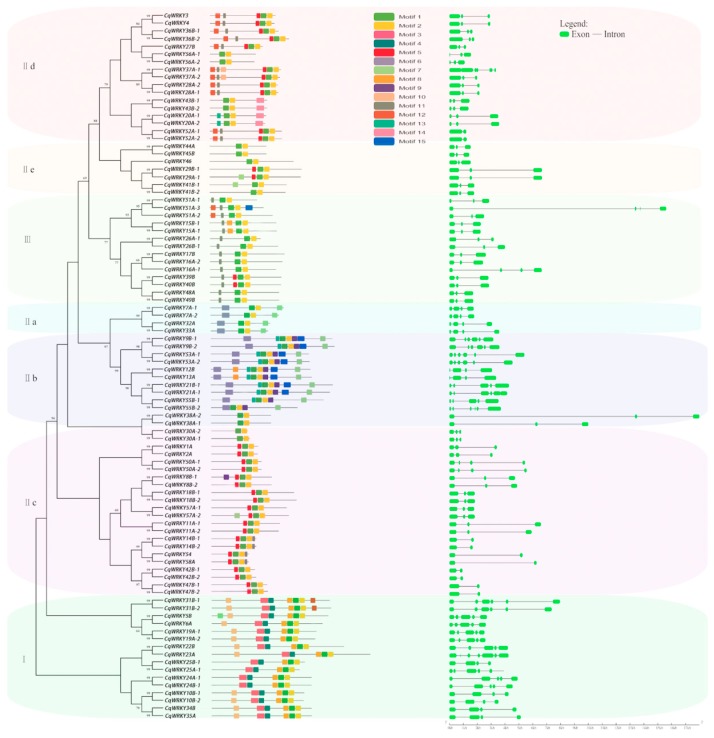
Motifs (**left**) and protein structure (**right**) of 92 CqWRKY proteins.

**Figure A11:**
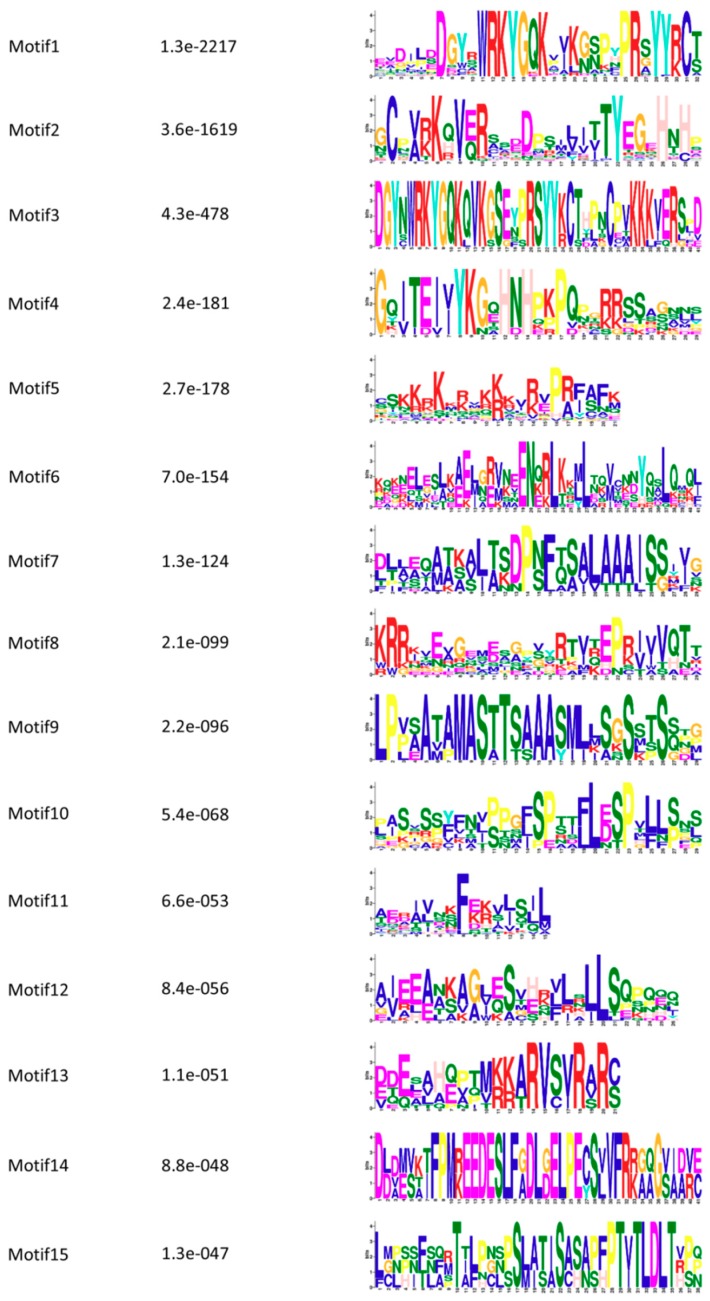
Logos of the CqWRKY protein conserved motifs. A total of 15 motifs were identified by the MEME (Multiple EM for Motif Elicitation) tool.

The conserved motifs of CqWRKY were examinedby using the MEME program. We identified 15 conserved motifs (Figure 3). The identified amino acids length of CqWRKY motifs ranged from 8 to 50. All sequence details of the conserved motifs are shown in Figure A11. The number of motifs was different in those proteins, ranging from two to eight. The results indicated that motif 1 was found in the WRKY domain, and motif 2 was defined as the zinc-finger domain. Similar motif compositions were found in the same group of CqWRKY proteins. Motif 7 was found in the group II-a and II-b subgroups and motif 13 in group III. In general, the location of introns and exons in the genome can provide important evidence for the evolutionary relationships of quinoa. The quinoa genome database was used to obtain the intron and exon distribution of CqWRKY. Results showed that the number of exons in CqWRKYs ranged from 2–6, and most contained 5 exons (Figure 2), while 88% of OsWRKY genes contained 2–6exons in rice. Among these genes, 47 OsWRKY genes (48%) contained 3exons [43]. For members of group II, the II-a and II-b subgroups contained 3–6 exons, group II-c contained 2 or 3 exons, and subgroups II-d and II-e contained 3 exons. All members of group III contained 3–4 exons (Figure 3). Similar to rice, 3 exon genes were the most common, but all of the group I OsWRKY genes contained more than 3 exons. Interestingly, it was surprising that three OsWRKY genes contained 1 exon and one OsWRKY gene contained 20 exons. In summary, this was the first time that the exons distributed in the CqWRKY were explored, which provide valuable information for the study of the evolutionary processes and expansion of the WRKY gene family in quinoa and other species.

### 3.6. Gene Duplication Analysis of CqWRKY

**Figure 4:**
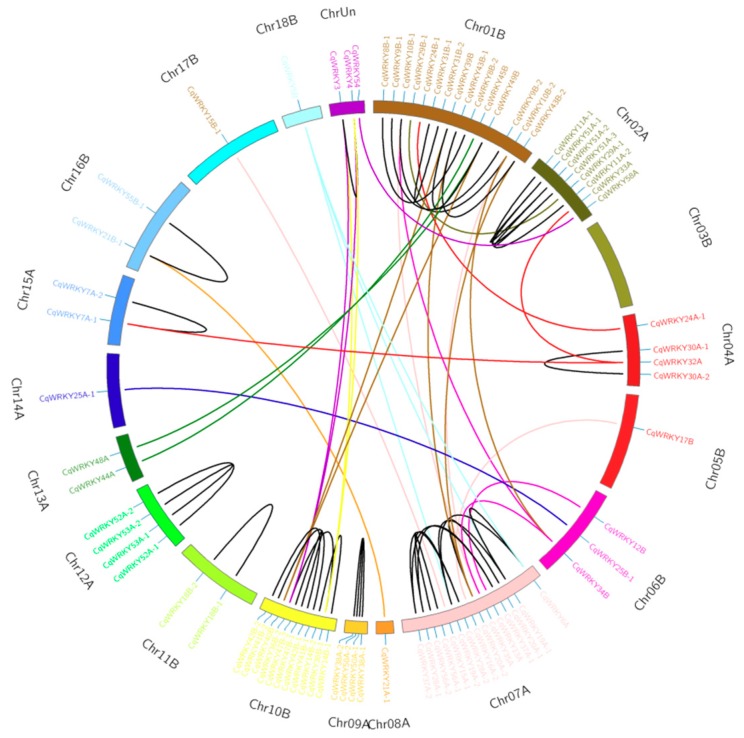
Duplicated CqWRKY genes pairs identified in quinoa. Quinoa chromosomes are shown in different colors. Duplicated gene pairs are linked by lines with different color.

Gene duplication, arising from polyploidization or during tandem and segmental duplication associated with replication, is a major factor causing gene family expansion [33,44]. In this study, 2 CqWRKY genes pairs, including CqWRKY51-1/2/3 and CqWRKY39B/40B/49B, were found to have three copies. 37 CqWRKY genes pairs contains two copies in the A and B homoeologous chromosome. Among them, the two copies of 25 genes pairs were existed together on the same chromosome. Just one copy of remaining 12 CqWRKY genes was identified in quinoa chromosomes by sequence similarity and chromosome localization (Figure 4). These results suggested gene loss may also happen in quinoa WRKY gene family, causing loss of some homologous copies.

### 3.7. Interaction Network between CqWRKY Genes and Other Genes in Quinoa

**Figure 5:**
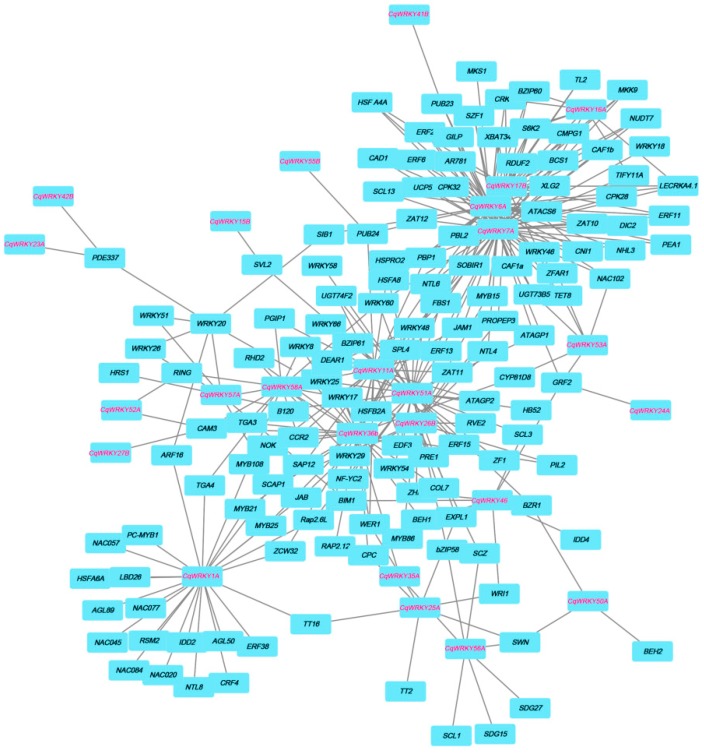
Network of CqWRKY genes in quinoa-based on the orthologues in *Arabidopsis*.

In order to understand the interaction relationship between CqWRKY and other genes, the co-expression network was created by an orthology-based method. We found 25 *CqWRKY* genes involved in 193 related genes of network interactions, including MYB, ZAT, NAC, ERF and WRKY gene family members (Figure 5). These results suggest that CqWRKY proteins maybe involved in a wide range of regulatory and stress related traits in quinoa. Previous studies have suggested that lateral organ boundaries domain (LBD) proteins are plant-specific transcription factors with a highly conserved LOB domain and play important roles in plant growth and development [45]. For example, LBD37, LBD38, and LBD39 not only act as novel repressors of anthocyanin biosynthesis and N availability signals, but also take part in metabolic regulation [46]. In the present study, CqWRKY1A was found to interact with LBD26 and other growth-related IDD2 genes. Furthermore, CqWRKYs were also involved in the response to abiotic stresses. For instance, CqWRKY51A, CqWRKY26B, and CqWRKY7A were found to interact with many quinoa stress-responsive genes including NAC102, ERF15, and MYB86/108 [47,48,49].

### 3.8. Expression Profile Analysis of CqWRKY Genes

**Figure 6:**
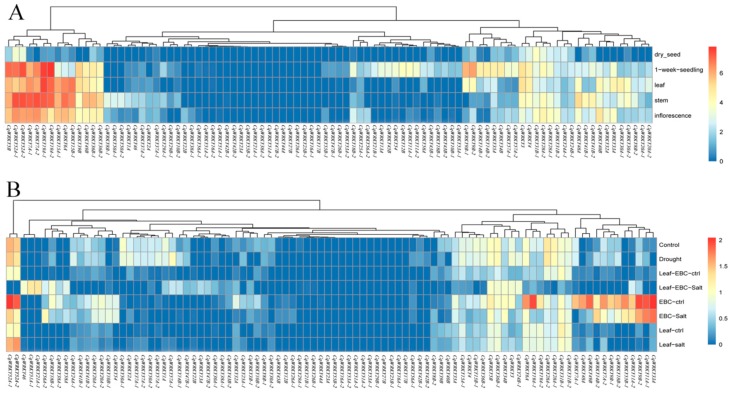
Relative expression level of 92 CqWRKY genes in specific tissues (**A**) and under different stresses (**B**). Red represents increased expression level.

In order to discover the tissue specificity and stress response genes in quinoa, the expression pattern of 92 CqWRKY genes were analyzed. RNA-seq data of various tissues including seedlings, stems, leaves, inflorescences, and seeds, and salt or drought stress were downloaded from the SRA database. The FPKM values were calculated by Hisat v2.0.4 (http://ccb.jhu.edu/software/hisat2/index.shtml) and Tophat v2.1.1 software (http://ccb.jhu.edu/software/tophat/index.shtml). Almost a quarter of the CqWRKY genes had no significant differences. Others have shown that clear tissue-specific expression. The CqWRKY gene family is involved in a wide range of growth and development processes in quinoa. Most CqWRKY shared a similar expression pattern between different copies in the A and B genome. *CqWRKY19A-1/2* in group III had the lowest expression levels in mature seeds, while the strongest expressions of these genes were detected in seedlings, stems, leaves, and inflorescences. In addition, the strongest expressions of *CqWRKY9B-1/2* were found in seedling and leaf, but showed low expression in other tissues. However, different pattern of *CqWRKY10B* expression was found between two copies in the B genome. *CqWRKY10B-1* showed strongest expression in 1-week seedling, while *CqWRKY10B-2* has the strongest expression in stem. These genes could be used as candidate genes for further functional studies (Figure 6A).

To study the stress response of CqWRKY, the expression level of each CqWRKY under salt or drought stress were analyzed. Almost half of the genes significantly induced expression under salt or drought stress, suggesting that these genes play an important role in response to abiotic stress. The expression levels of *CqWRKY52A-1/2* was similar in group II-d under salt stress was significantly higher than that of the control condition. The different copies of CqWRKY genes have different expression patterns in quinoa under abiotic stress. In particular, *CqWRKY21A-1* and *CqWRKY56A-2* had the highest expression levels under salt stress compared to normal conditions, while *CqWRKY21B-1* and *CqWRKY56A-1* had no significant expression difference, respectively (Figure 6B).

## 4. Discussion

The WRKY gene family plays vital roles in developmental process, diverse defense and abiotic stress responses [3,25,26]. Besides, WRKY proteins were not found in animal species and almost fungi species, while being widespread in land plants ranging from algae to angiosperm also make them fascinating candidates for the evolution of organism. The complex related features and biological functions of the WRKY gene family in *Arabidopsis* and rice have been extensively identified. Nevertheless, no data set of WRKY is available for quinoa. The WRKY gene family has been identified in 15 plant species by genome sequencing. We found that lower plants had smaller numbers of WRKY genes when compared to higher plants. Groups II-a and III were present in higher plants but not in ancient plants. This suggests an early evolution origin of these two groups in land plants. There were specific WRKY domain loss events in the evolution of WRKY from lower plants to higher plants. Groups II and III mainly regulate leaf senescence and responses to environmental stress. WRKY gene evolution might increase environment adaptability in higher plants.

In this study, using WRKY transcript factors of *Arabidopsis* as reference genes, we identified 92 CqWRKY genes in quinoa. The size of the WRKY gene family in quinoa was much higher than that of *Cucumis sativus* (57 members), grapevine (59 members), and *Hevea brasiliensis* (81 members), but lower than that of *Populus trichocarpa* (104 members), and rice (102 members) [27,50,51,52,53]. The abundance of transcription factor has been found to be largely dependent on sequence duplications during genome evolution [44]. The relatively high number of CqWRKY genes in quinoa indicates that the duplication events may have occurred during the genome evolution. It can be hypothesized that the presence of most WRKY genes in quinoa genome may reveal the specific requirements of these WRKY genes to be involved in the complicated mechanism of transcriptional regulation.

Additionally, 16, 62, and 14CqWRKY proteins were clustered into groups I, II, and III, respectively. Phylogenetic analysis showed that group I was considered as the original ancestor of the other two groups of the WRKY gene family, with structural variations and changes in the number of WRKY domains [54]. At the same time, the WRKYGQK heptapeptide variation and zinc finger structure changes also demonstrated the diversity of the evolutionary process of the WRKY gene family. Here, we found that the structure of the WRKY motif was highly conserved, and only seven CqWRKYs had the WRKYGQK mutation. Many species including broomcorn millet and wheat also showed a single amino acid variation [1,55]. Variance or variations in the WRKY motif can affect the normal activity of DNA binding. However, most closely related members in same subgroup share similar motif structure, also indicating these conserved motifs might have a similar role in subgroup functions (Figure 3).

Recent studies suggest that gene duplications not only play a vital role in the expansion and rearrangement of genome during the evolution process, but also induce gene function diversification [56,57]. Segmental duplication, tandem duplication, and transposition events are the three principal evolutionary patterns [58]. It has been reported that many gene duplications events had happened in apple, soybean and maize, which caused to the expansion of several gene classes [57,58,59]. Here, we found that among the 92 CqWRKY genes, 39 gene pairs duplication, consistent with previous report of gene duplication in quinoa (Figure 4) [21].

RNA-Seq data revealed the expression patterns of 92 CqWRKY genes in different tissues under drought and salt stress treatment [60,61]. The CqWRKY gene family in quinoa is involved in growth and development by RNA-seq data. Almost a third of CqWRKY genes had high expression in seedlings, stems, leaves, and inflorescences. Similar results were also found in poplar, *Salix suchowensis*, and cotton [62,63,64]. Interestingly, one CqWRKY gene (CqWRKY10B-1/2) has the different expression pattern between different copies in the B genome. Further studies are needed to determine the functions of the CqWRKY genes in quinoa (Figure 6A). Half of the CqWRKY genes were considered as stress response genes as these genes could be induced under salt and drought stress. CqWRKY18B-1, CqWRKY21A-1, CqWRKY51A-1 and CqWRKY56A-2 were highly regulated by salt stresses, suggesting that these genes might act as important stress regulators responses factors. Besides, the different copies of these CqWRKY genes have different expression patterns. CqWRKY18B-2 was significantly down-expressed under epidermal bladder cell (EBC)-salt stress. CqWRKY21B-1, CqWRKY51A-2 and CqWRKY51A-3, CqWRKY56A-1 was no difference under abiotic stress compared to normal conditions (Figure 6B).

## 5. Conclusions

Here, we studied the evolution analysis of the WRKY gene family in quinoa and other species, and systematically identified the CqWRKY gene family in quinoa at the genome-wide level. A total of 92 CqWRKY genes were identified. Based on the phylogenetic relationship and conserved motif and protein structure analysis, these genes were classified into three groups. We constructed the interaction network between CqWRKYs and other quinoa genes. A total of 193 gene interactions were identified. The CqWRKY genes were involved in a wide range of biological processes and stress responses in quinoa. These genes are good candidates for future functional analysis. These results will be beneficial for our understanding of the molecular mechanisms of stress tolerance in crops such as quinoa.

## Appendix A

**Table A1:** The detailed lists of 28 different species in this study.

Group	Number	Name	Group	Number	Name
Animal	1	*Echinococcus multilocularis*	Plant	1	*Marchantiapolymorpha*
	2	*Caenorhabditis elegans*		2	*Physcomitrella patens*
	3	*Drosophila melanogaster*		3	*Selaginellamoellendorffii*
	4	*Lates calcarifer*		4	*Pinus taeda*
	5	*Xenopus laevis*		5	*Piceaabies*
	6	*Callithrix jacchus*		6	*Arabidopsis thaliana*
	7	*Pan troglodytes*		7	*Solanum lycopersicum*
Fungi	1	*Rhizopus azygosporus*		8	*Brassica rapa*
	2	*Saccharomyces cerevisiae*		9	*Triticum aestivum*
	3	*Dictyostelium discoideum*		10	*Zea mays*
	4	*Gibberella*		11	*Oryza sativa*
	5	*Penicillium chrysogenum*		12	*Sorghum bicolo*
	6	*Ustilaginales*		13	*Chlamydomonas reinhardtii*
	7	*Uredinales*		14	*Volvox carteri*

**Table A2:** Characteristics of the putative WRKY proteins in quinoa.

Gene Name	Gene ID	Scaffold	Location	Protein Length	Subcellular localization	PI	MW(KDa)
CqWRKY1A	LOC110682067	2370	Chr09A	244	Nuclear	9.13	28.02363
CqWRKY2A	LOC110695228	3086	Chr07A	242	Nuclear	9.2	27.80931
CqWRKY3	LOC110682343	3452	ChrUn	343	Nuclear	9.46	37.58446
CqWRKY4	LOC110701719	3452	ChrUn	338	Nuclear	9.53	37.01582
CqWRKY5B	LOC110684199	2493	Chr18B	611	Nuclear	6.65	67.11943
CqWRKY6A	LOC110684669	2528	Chr07A	580	Nuclear	7.18	63.94286
CqWRKY7A-1	LOC110684974	2314	Chr15A	391	Nuclear	8.15	43.08997
CqWRKY7A-2	LOC110739966	2314	Chr15A	357	Nuclear	6.85	39.40957
CqWRKY8B-1	LOC110686140	1000	Chr01B	316	Nuclear	6.29	34.45764
CqWRKY8B-2	LOC110722117	1000	Chr01B	315	Nuclear	6.18	34.2994
CqWRKY9B-1	LOC110687395	1862	Chr01B	636	Nuclear	6.54	69.18332
CqWRKY9B-2	LOC110731310	1862	Chr01B	648	Nuclear	6.29	70.53042
CqWRKY10B-1	LOC110689002	2048	Chr01B	484	Nuclear	8.42	52.92818
CqWRKY10B-2	LOC110735049	2048	Chr01B	481	Nuclear	7.31	52.78703
CqWRKY11A-1	LOC110690337	1747	Chr02A	358	Nuclear	6.22	39.7074
CqWRKY11A-2	LOC110728618	1747	Chr02A	351	Nuclear	6.52	39.01257
CqWRKY12B	LOC110690435	2837	Chr06B	521	Nuclear	7.64	56.85428
CqWRKY13A	LOC110712087	1214	Chr07A	529	Nuclear	7.21	57.70618
CqWRKY14B-1	LOC110690523	1870	Chr10B	235	Nuclear	7.81	26.93543
CqWRKY14B-2	LOC110711270	1870	Chr10B	234	Nuclear	6.89	27.04852
CqWRKY15A-1	LOC110731563	1001	Chr07A	349	Nuclear	5.59	39.17953
CqWRKY15B-1	LOC110690718	2858	Chr17B	347	Nuclear	5.65	39.12759
CqWRKY16A-1	LOC110696174	1001	Chr07A	345	Nuclear	5.67	39.23355
CqWRKY16A-2	LOC110725677	1001	Chr07A	379	Nuclear	5.99	42.5395
CqWRKY17B	LOC110696200	3107	Chr05B	390	Nuclear	6.09	43.81166
CqWRKY18B-1	LOC110697304	2177	Chr11B	432	Nuclear	6.78	47.09947
CqWRKY18B-2	LOC110738211	2177	Chr11B	445	Nuclear	6.3	48.47655
CqWRKY19A-1	LOC110698105	1001	Chr07A	548	Nuclear	6	60.64106
CqWRKY19A-2	LOC110730454	1001	Chr07A	542	Nuclear	6	59.99832
CqWRKY20A-1	LOC110699703	3389	Chr07A	293	Nuclear	5.99	32.19663
CqWRKY20A-2	LOC110719121	3389	Chr07A	289	Nuclear	5.89	31.60399
CqWRKY21A-1	LOC110726496	1675	Chr08A	622	Nuclear	6.61	67.91068
CqWRKY21B-1	LOC110700550	3422	Chr16B	639	Nuclear	6.33	69.6783
CqWRKY22B	LOC110701089	3429	Chr05B	693	Nuclear	5.64	75.49235
CqWRKY23A	LOC110737209	2088	Chr07A	831	Nuclear	6.02	91.02864
CqWRKY24A-1	LOC110702356	1189	Chr04A	523	Nuclear	8.11	57.79983
CqWRKY24B-1	LOC110705651	3674	Chr01B	523	Nuclear	7.86	57.66874
CqWRKY25A-1	LOC110727210	1699	Chr14A	460	Nuclear	9.48	50.9117
CqWRKY25B-1	LOC110702616	3489	Chr06B	487	Nuclear	8.98	53.90871
CqWRKY26A-1	LOC110704369	3631	Chr04A	264	Nuclear	6.39	29.58899
CqWRKY26B-1	LOC110722941	1516	Chr01B	356	Nuclear	6.16	40.08192
CqWRKY27B	LOC110704656	3651	Chr16B	276	Nuclear	9.49	30.8523
CqWRKY28A-1	LOC110734403	2008	Chr07A	358	Nuclear	9.62	39.61248
CqWRKY28A-2	LOC110728935	2008	Chr07A	356	Nuclear	9.66	39.37821
CqWRKY29A-1	LOC110728527	1747	Chr02A	476	Nuclear	6.72	51.73296
CqWRKY29B-1	LOC110704717	1000	Chr01B	481	Nuclear	6.13	52.06432
CqWRKY30A-1	LOC110705289	3966	Chr04A	200	Nuclear	5.83	22.90195
CqWRKY30A-2	LOC110710397	3966	Chr04A	191	Extracellular	5.61	21.83396
CqWRKY31B-1	LOC110705952	3686	Chr01B	618	Nuclear	6.26	66.68202
CqWRKY31B-2	LOC110706951	3686	Chr01B	625	Nuclear	6.17	67.76825
CqWRKY32A	LOC110705979	1206	Chr04A	310	Nuclear	8.06	35.02528
CqWRKY33A	LOC110733669	1995	Chr02A	299	Nuclear	6.33	33.78876
CqWRKY34B	LOC110706383	3751	Chr06B	523	Nuclear	6.68	56.45296
CqWRKY35A	LOC110713207	1214	Chr07A	524	Nuclear	6.93	56.65928
CqWRKY36B-1	LOC110706462	1559	Chr10B	359	Nuclear	9.6	39.65634
CqWRKY36B-2	LOC110724341	1559	Chr10B	414	Nuclear	9.28	46.11367
CqWRKY37A-1	LOC110708385	2088	Chr07A	372	Nuclear	9.8	41.4642
CqWRKY37A-2	LOC110737126	2088	Chr07A	366	Nuclear	9.74	40.64239
CqWRKY38A-1	LOC110710092	2646	Chr09A	313	Nuclear	6.55	34.80783
CqWRKY38A-2	LOC110724156	2646	Chr09A	313	Nuclear	7.66	34.67071
CqWRKY39B	LOC110714054	4250	Chr01B	373	Nuclear	5.84	41.81242
CqWRKY40B	LOC110725171	1606	Chr10B	371	Nuclear	5.6	41.73932
CqWRKY41B-1	LOC110714254	1870	Chr10B	403	Nuclear	6.21	43.4019
CqWRKY41B-2	LOC110731586	1870	Chr10B	396	Nuclear	6.08	42.46192
CqWRKY42B-1	LOC110714280	1870	Chr10B	227	Nuclear	6.99	25.88965
CqWRKY42B-2	LOC110731590	1870	Chr10B	233	Nuclear	6.95	26.65745
CqWRKY43B-1	LOC110716147	4338	Chr01B	299	Nuclear	5.94	33.68204
CqWRKY43B-2	LOC110736290	4338	Chr01B	299	Nuclear	6	33.64207
CqWRKY44A	LOC110719591	1280	Chr13A	297	Nuclear	5.46	33.49528
CqWRKY45B	LOC110722558	1480	Chr01B	296	Nuclear	6.26	33.15403
CqWRKY46	LOC110729570	1783	ChrUn	438	Nuclear	5.92	48.43488
CqWRKY47B-1	LOC110719592	1870	Chr10B	292	Peroxisome	8.51	32.94533
CqWRKY47B-2	LOC110722559	1870	Chr10B	295	Peroxisome	8.11	33.28853
CqWRKY48A	LOC110719657	1280	Chr13A	361	Nuclear	5.83	40.4269
CqWRKY49B	LOC110722668	1480	Chr01B	361	Nuclear	5.79	40.31987
CqWRKY50A-1	LOC110720500	2370	Chr09A	262	Nuclear	8.71	29.83144
CqWRKY50A-2	LOC110721539	2370	Chr09A	262	Nuclear	8.26	29.9865
CqWRKY51A-1	LOC110720675	1529	Chr02A	247	Extracellular	5.37	28.26295
CqWRKY51A-2	LOC110723602	1529	Chr02A	329	Nuclear	5.84	37.03055
CqWRKY51A-3	LOC110723659	1529	Chr02A	281	Nuclear	5.27	32.04714
CqWRKY52A-1	LOC110720830	1373	Chr12A	376	Nuclear	9.58	41.44137
CqWRKY52A-2	LOC110726911	1373	Chr12A	377	Nuclear	9.54	41.6516
CqWRKY53A-1	LOC110720931	1373	Chr12A	513	Nuclear	5.55	56.43854
CqWRKY53A-2	LOC110723870	1373	Chr12A	517	Nuclear	5.46	57.13047
CqWRKY54	LOC110723391	3819	ChrUn	195	chloroplast	9.43	22.49956
CqWRKY55B-1	LOC110723405	1526	Chr16B	589	Nuclear	6.13	64.4822
CqWRKY55B-2	LOC110727007	1526	Chr16B	452	Nuclear	7.98	49.25958
CqWRKY56A-1	LOC110732902	2008	Chr07A	239	Nuclear	9.05	27.35754
CqWRKY56A-2	LOC110734287	2008	Chr07A	232	Nuclear	8.94	26.56771
CqWRKY57A-1	LOC110733008	1992	Chr09A	393	Nuclear	6.42	42.77934
CqWRKY57A-2	LOC110734264	1992	Chr09A	405	Nuclear	6.26	43.97958
CqWRKY58A	LOC110739691	2306	Chr02A	194	Cytoplasm	9.5	22.37437
